# Building a global schistosomiasis alliance: an opportunity to join forces to fight inequality and rural poverty

**DOI:** 10.1186/s40249-017-0280-8

**Published:** 2017-03-23

**Authors:** Lorenzo Savioli, Marco Albonico, Daniel G. Colley, Rodrigo Correa-Oliveira, Alan Fenwick, Will Green, Narcis Kabatereine, Achille Kabore, Naftale Katz, Katharina Klohe, Philip T. LoVerde, David Rollinson, J. Russell Stothard, Louis-Albert Tchuem Tchuenté, Johannes Waltz, Xiao-Nong Zhou

**Affiliations:** 1 Global Schistosomiasis Alliance, Geneva, Switzerland; 2 Center for Tropical Diseases, Sacro Cuore Hospital - WHO Collaborating Centre on strongyloidiasis and other intestinal parasitic infections, Negrar, Verona Italy; 3 Schistosomiasis Consortium for Operational Research and Evaluation, The University of Georgia, Athens, Georgia USA; 4 Centro de Pesquisas René Rachou – Fiocruz, Belo Horizonte, Brazil and Universidade Federal de Ouro Preto, Ouro Preto, Brazil; 5 Department of Infectious Disease Epidemiology, SCI, Imperial College, London, UK; 6 Trinity College Cambridge, Cambridge, UK; 7 Imperial College London, London, UK; 8 RTI International, Washington, DC, USA; 9 Research Center René Rachou – Oswaldo Cruz Foundation, Belo Horizonte, Brazil; 10 Global Schistosomiasis Alliance, Munich, Germany; 11 University of Texas Health Science Center, San Antonio, TX USA; 12 Life Sciences Department, The Natural History Museum, London, UK; 13 Department of Parasitology, Liverpool School of Tropical Medicine, Liverpool, L3 5QA UK; 14 Centre for Schistosomiasis & Parasitology, Yaounde, Cameroon; 15 Global Schistosomiasis Alliance, London, UK; 16 National Institute of Parasitic Diseases Chinese Center for Disease Control and Prevention, Shanghai, China

**Keywords:** Neglected tropical diseases, Schistosomiasis, Global Schistosomiasis Alliance, Elimination

## Abstract

Schistosomiasis, one of the 17 neglected tropical diseases listed by the World Health Organization, presents a substantial public health and economic burden. Of the 261 million people requiring preventive chemotherapy for schistosomiasis in 2013, 92% of them lived in sub-Saharan Africa and only 12.7% received preventive chemotherapy. Moreover, in 2010, the WHO reported that schistosomiasis mortality could be as high as 280 000 per year in Africa alone.

In May 2012 delegates to the sixty-fifth World Health Assembly adopted resolution WHA65.21 that called for the elimination of schistosomiasis, and foresees the regular treatment of at least 75% of school age children in at-risk areas. The resolution urged member states to intensify schistosomiasis control programmes and to initiate elimination campaigns where possible.

Despite this, in June 2015, schistosomiasis was indicated to have the lowest level of preventive chemotherapy implementation in the spectrum of neglected tropical diseases. It was also highlighted as the disease most lacking in progress. This is perhaps unsurprising, given that it was also the only NTD with access to drug donations but without a coalition of stakeholders that collaborates to boost commitment and implementation.

As a consequence, and to ensure that the WHO NTDs Roadmap Targets of 2012 and World Health Assembly Resolution WHA65.21 are met, the Global Schistosomiasis Alliance (GSA) has been set up. Diverse and representative, the GSA aims to be a partnership of endemic countries, academic and research institutions, international development agencies and foundations, international organizations, non-governmental development organizations, private sector companies and advocacy and resource mobilisation partners. Ultimately, the GSA calls for a partnership to work for the benefit of endemic countries by addressing health inequity and rural poverty.

**Electronic supplementary material:**

The online version of this article (doi:10.1186/s40249-017-0280-8) contains supplementary material, which is available to authorized users.

## Multilingual abstracts

Please see Additional file 1 for translations of the abstract into the five official languages of the United Nations.

## Introduction

Schistosomiasis is one of 17 diseases that are listed by the World Health Organization (WHO) as the group of neglected tropical diseases (NTDs). Of these, schistosomiasis presents a substantial public health and economic burden. In 2010, the WHO reported that schistosomiasis mortality could be as high as 280 000 per year in Africa alone [1]. Schistosomiasis is also considered a disease of poverty [2]. For example, in southwestern Nigeria the prevalence of *Schistosoma haematobium* infection increases from 1.5% for households with an income greater than US $1 600, to 70% for households with an income of less than US $600 [3]. Specifically, incidence of infection is concentrated in particularly poor communities with a dependence on surface water, water that is often contaminated with urine and faeces of infected individuals, and colonised by snails that act as the intermediate hosts for the schistosome [4]. Individuals, especially children, become infected and re-infected by regular exposure to the contaminated water [5]. Due to this relationship with poverty and water, the distribution of schistosomiasis in Africa remains highly widespread and yet highly focal at the local level [6].

Praziquantel (PZQ) is the drug of choice for effective treatment of schistosomiasis [7]. Developed in the 1970s by Merck and Bayer AG in Germany [8], it generated hope for a fast reduction in morbidity in sub-Saharan Africa where morbidity control had not developed as in other endemic countries. The drug is easy to administer by the aid of a dose pole for height [2], presenting extremely good efficacy in a single dose, and can be safely administered during pregnancy and lactation [9]. Treating infected individuals could thus be straightforward. However, despite the drug’s relatively low price, its limited availability has long hindered progress towards control, particularly in sub-Saharan Africa. Subsequently, at the population-level many communities in endemic areas still lack sufficient access to treatment. Of the 261 million people requiring preventive chemotherapy for schistosomiasis in 2013, 92% of them lived in sub-Saharan Africa and globally only 12.7% received treatment by means of preventive chemotherapy (PC), despite on-going PC campaigns [10].

There has been progress since, with 20.7% of those requiring treatment receiving it in 2014 (equivalent to 61.6 million people) and a further increase to 28% (equivalent to 65.2 million people of which 52.7 million represent children) in 2015 [11]. Furthermore, in some former endemic countries in Asia, Latin America and the Middle East, the disease has been controlled or even eliminated, demonstrating the feasibility of fighting schistosomiasis. In China for example, Chairman Mao Zedong made schistosomiasis a public health priority in the 1950s as its control was seen to be a critical element for rural development [12–16]. Brazil was also able to achieve significant reductions in morbidity [16].

In many cases, however, a lack of both political will and committed resources remains. For example, only a few countries in Africa, despite recent increases in GDP [17] have committed national resources to fighting schistosomiasis and “scale-up remains slow in the highest burden countries where 70% of the burden occurs” [12]. Consequently, in many parts of Africa, the expansion of control programmes is progressing too slowly and schistosomiasis continues to be transmitted in many rural areas, hindering economic and human development [18]. Importantly, this seems to be the case despite a growing interest in fighting neglected tropical diseases in general and schistosomiasis in particular.

With the understanding of the cost-effectiveness of PC interventions to control or, if associated with other interventions, support the elimination of schistosomiasis and other NTDs came the commitments of major pharmaceutical manufacturers to provide the necessary medicines free of charge. In 2012 a number of large pharmaceutical manufacturers have pledged to continue or extend large medicinal donations under the London Declaration on NTDs. The cumulative value of these donations of US $17.8 bn from 2014–2020, represents the greatest growing public health donation and additionally targets the most vulnerable afflicted populations in the world [12]. One of these donations comes from the science and technology company Merck that has made a commitment to donate PZQ until schistosomiasis has been eliminated. As a signatory of the London Declaration on NTDs Merck has committed to a ten-fold increase in its annual donation of PZQ (a step-wise increase from 25 million to up to 250 million tablets equivalent to 100 million doses a year) with a focus on African school-age children [13].

Yet, in addition to improved supply of the drug, there is a need for research and development into, amongst others, combination therapies [14], and biomarkers monitoring the emergence of drug resistance, as well as implementation and distribution strategies and the revitalisation of previously used anthelmintics [15]. This is particularly important as PZQ currently remains the only asset in the chemotherapy armamentarium against schistosomiasis. Furthermore, since schistosomiasis is known to rapidly proliferate in badly planned irrigation systems or dam structures [19], reducing infection and transmission of the disease by means additional to access to free regular anthelminthic treatment such as snail control and properly organized water and sanitation plans, is critical for equitable agricultural development schemes.

Considering this potential for real impact thanks to the growing interest in and commitments to schistosomiasis and at the same time the many unmet needs and open questions, the purpose of this review is to highlight the need of having an alliance that addresses the global elimination of schistosomiasis by aligning these two sides of the coin.

## The need for an alliance

In May 2012 delegates to the sixty-fifth World Health Assembly adopted resolution WHA65.21 that called for the elimination of schistosomiasis [20]. Previous resolutions and the WHO NTD Road Map of 2012 had foreseen the regular treatment of at least 75% of school age children in at-risk areas as the basis for morbidity and disease control [21–23]. The elimination of schistosomiasis resolution of 2012 urged member states to intensify schistosomiasis control programmes and to initiate elimination campaigns where possible, through strengthened health systems, preventive chemotherapy, and provision of water and sanitation as well as hygiene, education and snail control.

A central problem for the achievement of the above resolution, however, is the fact that, until now, the schistosomiasis community has been built mainly around separate research-driven activities. These include, but are not limited to, the EU funded CONTRAST programme, the Bill & Melinda Gates Foundation funded Schistosomiasis Consortium for Operational Research and Evaluation (SCORE) programme as well as The Schistosomiasis Control Initiative (SCI), based at Imperial College London, which has been the major body dealing with implementation in Africa. Most recently, the Research and Evidence Division of DFID has invested in COUNTDOWN, a multidisciplinary implementation research consortium engaged in identifying best ways to foster scale-up of preventive chemotherapy approaches in sub-Saharan Africa.

While each organisation and its efforts are very valuable, coordination and communication amongst the stakeholders involved has so far been limited. According to the authors, this lack of a coordinated approach to the control and elimination of schistosomiasis as a public health problem was reflected in the poor performance of the disease vis-a-vis other NTDs in the scorecard of the 3^rd^ annual progress report published by Uniting to Combat NTDs. [24]. Schistosomiasis lagged behind, for example, onchocerciasis, soil-transmitted helminthiasis and trachoma, which have been benefiting from a partnership of organisations.

The experience of previous NTD alliances shows that they provide a necessary platform for keeping a committed community together, whilst being able to highlight operational research needs and freely advocate for stronger political commitment. A significant change in the organisation of efforts is hence considered necessary to ensure that the increased donation from Merck is used effectively in endemic country settings in Africa if the WHO NTDs Roadmap Targets of 2012, and World Health Assembly Resolution WHA65.21, are to be met. Maintaining or expanding efforts in the rest of the world will need sustainable political and financial commitment by countries where transmission has reached the critical point towards elimination. Meeting these targets will require building a robust coalition of a multitude of stakeholders and securing stronger political commitment in endemic countries especially, but not exclusively, in Africa.

## Building the Global Schistosomiasis Alliance

Such a coalition has now been established in the form of the Global Schistosomiasis Alliance (GSA) in response to the challenge afforded by WHA65.21 and in a manner similar to other NTD coalitions [25].

The major aim of the GSA is to be a partnership of endemic countries, academic and research institutions, international development agencies and foundations, international organizations, non-governmental development organizations, private sector companies and advocacy and resource mobilisation partners. Thereby, the GSA can communicate with and between all different stakeholders working on and engaged with schistosomiasis. While a number of key stakeholders have already become member of the GSA, the GSA’s aim is to capitalise upon the growing momentum for control and elimination of schistosomiasis and unite a growing number of members who all work towards the objectives outlined in WHA65.21. In particular, the GSA would welcome if an increasing number of African based organisations and institutions, as well as African governments were to join the alliance.

The GSA is conceptualised as an independent organisation allowing for transparency, as well as allowing all members to speak openly and independently. Furthermore, this organisational set-up respects the autonomy of each partner and promotes efficient and effective governance. Thereby the GSA is hoping to create a space that increases engagement and alignment among partners and optimises the contribution of alliance members for the greatest possible impact.

**Fig. 1:**
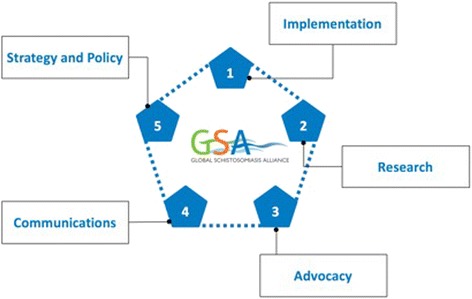
GSA Priority Areas

With elimination as the overarching objective, the work of the GSA focuses on five interconnected themes, presented below in Fig. 1.

The immediate focus lies on priority number one, implementation. This refers to the provision of support to the scale-up of preventive chemotherapy interventions and other operational components for the control and elimination of schistosomiasis based on successes already achieved and lessons learned in several countries all over the world. The communication of best practices between countries and stakeholders and capacity building are considered central tasks of the GSA.

Secondly, research is an important part of the GSA’s strategy towards elimination. Specific research areas to be addressed, as well as research activities the GSA has engaged in so far through its research working group are outlined below in more detail. A third priority of the GSA is advocacy work, both in endemic and non-endemic countries, which will be coordinated with key regional stakeholders. The aim is to raise awareness of the importance to address schistosomiasis and to mobilise resources together with GSA stakeholders. This links directly to the fourth priority, which concerns communications. In order to deliver its key messages, the GSA employs a variety of communication channels and tools, such as newsletters and campaigns, to reach and target stakeholders. The “Something in the Water” website (http://sitw.eliminateschisto.org/int/) is one such campaign, that reached viewers globally and won six awards in 2016 for its creative approach. Finally, as the GSA becomes more established, it will use its influence to continue to push its mission to eliminate schistosomiasis as a public health problem. The goal is to do so via a number of different actions, such as arriving at one common and shared definition of elimination and the required actions to achieve it, coordinating and commissioning global research efforts, as well as close collaboration with WHO to ensure optimal allocation of resources that support schistosomiasis elimination.

The latter will also be addressed by cooperation and collaboration with the STH Coalition, due to connections of STH and schistosomiasis. The target set by WHO in the NTD Road map for both STH and schistosomiasis is a minimum 75% coverage with PC for all school age children to be reached by 2020 [21, 22]. STH and schistosomiasis are also often found to be co-occurring in the same communities [2] and shared treatment and medication distribution as well as monitoring and evaluation are important aspects to be considered.

A number of working groups have been or will be set up, covering the above mentioned strategically important aspects. Details regarding these working groups can be found in the table below Table 1.

**Table 1:** Research Working Groups supporting GSA activities

1.	Research and development
2.	Implementation (enhance and support country programmes through, e.g., project facilitation, technical support, capacity building and training)
3.	Monitoring and Evaluation
4.	Promotion of quality control, supply and distribution of PZQ
5.	Advocacy and resource mobilisation
6.	Enhancement of collaboration and coordination with other NTD PC programmes
7.	Support of IEC (information, education & communication) activities and community issues

The tasks of these working groups are to address the areas where there is still much to learn, as for example about the diagnostics needed in low transmission settings and complementary interventions such as snail control, behaviour change and water supply and sanitation, through novel educational programmes targeting mainly children. Moreover, the GSA and its members are engaged in or support research into the development of a paediatric formulation of PZQ [26], the relationship between urogenital and female genital schistosomiasis and HIV [27–29] and drug resistance [30, 31]. The importance of collaborating with other NTDs is highlighted by a recent article that discusses significant associations between HIV infection, schistosomiasis and lymphatic filariasis in terms of the host’s Th1- and Th2-type immune responses [32].

The research and development working group is a good example of activities so far, as it organised a conference in Shanghai in June, 2016, in collaboration with the National Institute of Parasitic Diseases, Chinese Center for Disease Control and Prevention, entitled “Schistosomiasis Research: Providing the Tools Needed for Elimination”, from which this Special Edition is derived. Among the general but concrete outcomes of necessary steps to take that all participants agreed on were the need for the development of clearer alternative schedules for the use of preventive chemotherapy and new guidelines for the elimination of schistosomiasis with measurable recommendations for WASH as well as specific recommendations for snail control and the management of persistent hot-spots.

The implementation working group is currently playing an important role in the establishment and coordination of a multi-stakeholder elimination project in Ethiopia. The GSA has been bringing in different stakeholders, donors and the Ministry of Health and coordinates amongst all partners to develop and monitor an elimination approach. The aim is to document the approach, with its strengths and weaknesses in such a way that it can be used for and applied to other settings. As part of the implementation working group, effective monitoring and evaluation approaches have also already been targeted by the GSA. It is currently working towards a revised set of programme support milestones for the Uniting to Combat Scorecard to allow for better tracking of the progress that is being made and highlighting the areas that require more attention.

In all its activities, the GSA is aware of the importance not to duplicate any efforts or take over the responsibilities of others. The GSA also considers it to be critical to ensure that the schistosomiasis community recognises the distinction between the programmatic responsibilities of the WHO (which provide technical and strategic advice to endemic countries) and member states of the WHO, that collaborate with non-governmental development organizations (NGDOs), co-operate with non-endemic member states through bilateral agreements and are responsible for distribution of medicines in the country. Furthermore, most donations, including that of PZQ, are based upon a memorandum of understanding between the WHO and the specific pharmaceutical company. The GSA supports this set-up since it facilitates coordination, forecasting and procurement by member states of the WHO.

## Conclusion

Ultimately the GSA is working for the benefit of endemic countries and providing support on the way to schistosomiasis elimination. In turn, the growing economies of Africa will need to invest in schistosomiasis elimination programmes in sustained ways. This will demonstrate their commitment to reaching the sustainable development goals by 2030, to develop their rural communities in an equitable way and to reduce the burden of poverty in their countries. By uniting highly committed stakeholders the GSA aims to support these African countries by facilitating partnerships, which address health inequities, reduce rural poverty, and work towards the elimination of schistosomiasis for the benefit of the world’s most vulnerable communities with the ultimate objective to assure equitable human, agricultural and economic development.

## Additional file

Additional file 1: Multilingual abstracts in the five official languages of the United Nation. (PDF 656 kb)

## Abbreviations

GSA: Global Schistosomiasis Alliance
IEC: information, education and communication
NTDs: Neglected tropical diseases
PC: Preventive chemotherapy
PZQ: Praziquantel
SCI: Schistosomiasis control initiative
SCORE: Schistosomiasis consortium for operational research and evaluation
WHO: World Health Organization
