# Supplementary material for: Building a global schistosomiasis alliance: an opportunity to join forces to fight inequality and rural poverty
Source: Infect Dis Poverty. 2017 Mar 23;6:65. doi: 10.1186/s40249-017-0280-8 (PMC5363045; doi:10.1186/s40249-017-0280-8)
Supplement: Additional file 1: — Multilingual abstracts in the five official languages of the United Nation. (PDF 656 kb) [file 40249_2017_280_MOESM1_ESM.pdf]

Translation of the abstract into the six official working languages of the United Nations

بناء جمعية عالمية لداء البلهارسيا (الشستوسوما): فرصة للتضامن ومحاربة عدم المساواة والفقر في المناطق النائية

Lorenzo Savioli, Marco Albonico, Daniel G. Colley, Rodrigo Correa-Oliveira, Alan Fenwick, Will Green, Narcis Kabatereine, Achille Kabore, Naftale Katz, Katharina Klohe, Philip T. LoVerde, David Rollinson, J. Russell Stothard, Louis-Albert Tchuem Tchuenté, Johannes Waltz, Xiao-Nong Zhou

ملخص

داء البلهارسيا، أحد الأمراض في قائمة "الأمراض المدارية المهملة" لدى منظمة الصحة العالمية، يشكل عبئا كبيرا على الصحة العامة والاقتصاد. في عام 2013، احتاج 261 مليون شخص لعلاج دوائي وقائي، 92% منهم يعيشون في قارة إفريقيا جنوب الصحراء الكبرى، ولم يتلقى العلاج سوى 12,7% من هؤلاء. بالإضافة، في عام 2010، أعلنت منظمة الصحة العالمية أن معدل الوفيات من داء البلهارسيا قد يصل إلى 280000 ألف وفاة سنوياً في قارة إفريقيا فقط.

وفي مايو من عام 2012، تبنّى ممثّلي الاجتماع الخامس والستين لجمعية الصحة العالمية قرار WHA65.21 والذي طالب بالقضاء على داء البلهارسيا، والذي يُشرف على العلاج المنتظم لـ 75% من الأطفال في عمر المدارس في المناطق المعرّضة للمرض. وقد حثّ القرار الدول الأعضاء على تكثيف برامج السيطرة على عدوى البلهارسيا بالإضافة إلى بدء حملات للقضاء عليه أينما تكون ممكنة.

على الرغم من ذلك، ففي يونية من عام 2015، وُجد أن داء البلهارسيا كان الأقل نسبة فيما يتعلق بتطبيق العلاج الوقائي من بين الأمراض المدارية المهملة الأخرى. كما اتضح أنه أكثرها افتقارا لإحراز تقدما بهذا الخصوص. وقد لا يكون ذلك مستغربا، لكونه المرض الوحيد من ضمن مجموعة الأمراض المدارية المهملة التي لم يجد متبرعين مهتمين بتعزيز الاهتمام بأمره وتطبيق العلاج مع توافر العلاج المتبرع.

ونتيجة لذلك، ولضمان تنفيذ أهداف خارطة الأمراض المدارية المهملة 2012 التي وضعتها منظمة الصحة العالمية وقرار WHA65.21 لجمعية الصحة العالمية، فقد تم تأسيس الجمعية العالمية للبلهارسيا. تهدف الجمعية العالمية للبلهارسيا، والتي تشمل عناصر متنوعة وممثلة للقطاعات، لتكون شراكة بين الدول التي يستوطن فيها المرض، والمؤسسات الأكاديمية ومراكز الأبحاث، والمؤسسات التنموية العالمية، والمنظمات العالمية، والمؤسسات التنموية غير الحكومية، وشركات القطاع الخاص وشركاء حشد الموارد والمناصرة. وفي النهاية تطالب الجمعية العالمية للبلهارسيا بشراكة لأجل العمل لصالح الدول التي يستوطن فيها المرض من خلال مواجهة عدم المساواة في الأمور الصحية والفقر في المناطق النائية.

Translated from English version into Arabic by SAlkhodair, through

TRANSLATORS
WITHOUT BORDERS

建立全球血吸虫病联盟：合力应对卫生不公和农村贫困

Lorenzo Savioli, Marco Albonico, Daniel G. Colley, Rodrigo Correa-Oliveira, Alan Fenwick, Will Green, Narcis Kabatereine, Achille Kabore, Naftale Katz, Katharina Klohe, Philip T. LoVerde, David Rollinson, J. Russell Stothard, Louis-Albert Tchuem Tchuenté, Johannes Waltz, Xiao-Nong Zhou

摘要

血吸虫病是世界卫生组织（WHO）列出的 17 种被忽视的热带病（NTD）之一，造成了巨大的公共卫生和经济负担。2013 年，有 2.61 亿血吸虫病患者需要预防性化疗，其中 92%生活在撒哈拉以南非洲地区，但只有 12.7%接受了预防性化疗。此外，据世卫组织 2010 年报告称，非洲血吸虫病死亡数每年可高达 280 000。

2012 年 5 月，第六十五届世界卫生大会的代表通过了 WHA65.21 号决议，号召消除血吸虫病；预见在风险地区至少 75%的学龄儿童能得到定期治疗。该决议敦促成员国强化血吸虫病控制项目实施，并在可能的情况下启动消除行动。
尽管如此，2015 年 6 月，在被忽视的热带疾病谱系中，针对血吸虫病实施的预防性化疗水平被指最低，其防控工作也最滞后。这也许并不足为奇，因为血吸虫病是唯一一个获得药物捐赠的 NTD，但它却没有一个利益相关者联盟合作来提高承诺和促进项目实施。
因此，各利益相关者建立全球血吸虫病联盟（GSA），确保实现“2012 年世卫组织 NTDs 路线图目标”和“世界卫生大会 WHA65.21 号决议”条款。GSA 旨在成为流行国家、学术和研究机构、国际发展机构和基金会、国际组织、非政府发展组织、私营业公司以及宣传和资源调动合作伙伴中具有多样性和代表性的一员。最终，GSA 呼吁建立合作伙伴关系，解决卫生不公平和农村贫困，为血吸虫病流行国家的利益服务。

Translated from English version into Chinese by Jin Chen, edited by Pin Yang

CHINESE CENTER FOR DISEASE CONTROL AND PREVENTION
NATIONAL INSTITUTE OF PARASITIC DISEASES

Création d’une Alliance Mondiale contre la Schistosomiase: L’occasion d’unir ses forces pour lutter contre les inégalités et la pauvreté rurale.

Lorenzo Savioli, Marco Albonico, Daniel G. Colley, Rodrigo Correa-Oliveira, Alan Fenwick, Will Green, Narcis Kabatereine, Achille Kabore, Naftale Katz, Katharina Klohe, Philip T. LoVerde, David Rollinson, J. Russell Stothard, Louis-Albert Tchuem Tchuenté, Johannes Waltz, Xiao-Nong Zhou

Résumé

La schistosomiase, une des 17 maladies tropicales négligées sur la liste de l’Organisation Mondiale de la Santé, constitue un fardeau considérable pour la santé publique et l’économie. Sur les 261 millions de personnes nécessitant une chimiothérapie préventive de la schistosomiase en 2013, 92% vivent en Afrique sub-saharienne, et seulement 12,7% ont reçu une chimiothérapie préventive. En outre, en 2010, l’OMS a indiqué que les cas de mortalité causés par la schistosomiase pourraient s’élever à 280 000 par an en Afrique seule.
En Mai 2012, les représentants de la 65ème Assemblée Mondiale de la Santé ont adopté la résolution WHA65.21, qui a appelé à l’élimination de la schistosomiase, et qui prévoit le traitement régulier d’au moins 75% d’enfants en âge scolaire dans les régions à risque. La résolution a exhorté les états membres à intensifier les programmes de surveillance de la schistosomiase et de commencer les campagnes d’élimination là où cela est possible.
Malgré cela, en Juin 2015, la schistosomiase s’est avérée avoir le plus bas niveau de mise en œuvre de chimiothérapie préventive dans le spectre des maladies tropicales négligées. Cette maladie a également été signalée comme étant celle qui manquait le plus d’amélioration. Ce n’est peut-être pas surprenant, étant donné que c’était aussi la seule MTN ayant accès aux dons de médicaments, mais sans groupement d’intervenants collaborant à intensifier l’engagement et la mise en œuvre.
En conséquence, et pour veiller à ce que les Cibles de la Feuille de Route de 2012 de l’OMS pour les MTN, et de la Résolution WHA65.21 de l’Assemblée Mondiale de la Santé soient respectées, l’Alliance Mondiale contre la Schistosomiase (AMS) a été créée. Diverse et représentative, l’AMS a pour objectif d’être un partenariat des pays endémiques, des institutions d’enseignement et de recherche, des fondations et des organismes internationaux de développement, d’organismes internationaux, d’organismes de développement non-gouvernementaux, des sociétés du secteur privé et de mobilisation, et partenaires de mobilisation de ressources. Finalement, l’AMS appelle à un partenariat

en vue de travailler pour le bénéfice des pays endémiques en s'attaquant aux inégalités en matière de santé et de pauvreté rurale.

Translated from English version into French by Ode Laforge, through

TRANSLATORS
WITHOUT BORDERS

Создание Глобального альянса по борьбе с шистосомозом: возможность объединить усилия для борьбы с неравенством и бедностью в сельских районах

Lorenzo Savioli, Marco Albonico, Daniel G. Colley, Rodrigo Correa-Oliveira, Alan Fenwick, Will Green, Narcis Kabatereine, Achille Kabore, Naftale Katz, Katharina Klohe, Philip T. LoVerde, David Rollinson, J. Russell Stothard, Louis-Albert Tchuem Tchuenté, Johannes Waltz, Xiao-Nong Zhou

Краткий обзор

Шистосомоз, одна из 17 «забытых» тропических болезней, входящих в перечень Всемирной Организации Здравоохранения, представляет собой значительное бремя для общественного здравоохранения и экономики. Из 261 миллиона человек, нуждающихся в лечении профилактической химиотерапией против шистосомоза в 2013 году, 92% проживали в странах Африки, расположенных к югу от Сахары, и только 12,7% получили лечение профилактической химиотерапией. Кроме того, в 2010 году, по оценкам ВОЗ, только в Африке смертность от шистосомоза составила более 280 000 человек.

В мае 2012 года делегаты 65-ой сессии Всемирной Ассамблеи Здравоохранения приняли резолюцию WHA65.21, призывающую к ликвидации шистосомоза и предполагающую регулярное лечение как минимум 75% детей школьного возраста в районах, подвергающихся особому риску. Резолюция призвала государства-члены усилить программы по контролю шистосомоза и, где такая возможность существует, инициировать программы по его ликвидации.

Несмотря на это, в июне 2015 года, шистосомоз был признан болезнью с наименьшим уровнем охвата населения профилактической химиотерапией среди всех заболеваний, относящихся к «забытым» тропическим болезням. Также он был отмечен, как заболевание с наименьшим уровнем прогресса. Это, пожалуй, и неудивительно, если принять во внимание тот факт, что это была единственная ЗТБ, имеющая доступ к пожертвованным лекарствам, но без коалиции заинтересованных сторон, работающих вместе для увеличения уровня вовлеченности и охвата населения.

Как следствие, с целью обеспечения выполнения целей дорожной карты ВОЗ по ЗТБ в 2012 году и резолюции WHA65.21 Всемирной Ассамблеи Здравоохранения, был основан Глобальный альянс по борьбе с шистосомозом (GSA, Global Schistosomiasis Alliance). Многообразный и представительный, альянс ставит перед собой цель установить партнерство с участием эндемичных стран, научно-исследовательских организаций, международных агентств по развитию и фондов, международных организаций, неправительственных организаций по развитию, компаний из частного сектора и партнеров по информационно-пропагандистской деятельности и мобилизации

ресурсов. Как итог, альянс призывает к партнерству, направленному на работу на благо эндемичных стран, концентрируя усилие на борьбу с неравенством в сфере здравоохранения и бедности в сельских районах.

Translated from English version into Russian by Dmitry Esin

Desarrollo de una alianza global contra la esquistosomiasis: Una oportunidad para aunar fuerzas para combatir la desigualdad y la pobreza rural

Lorenzo Savioli, Marco Albonico, Daniel G. Colley, Rodrigo Correa-Oliveira, Alan Fenwick, Will Green, NarcisKabatereine, AchilleKabore, Naftale Katz, Katharina Klohe, Philip T. LoVerde, David Rollinson, J. Russell Stothard, Louis-Albert TchuemTchuenté, Johannes Waltz, Xiao-Nong Zhou

Resumen

La esquistosomiasis, una de las diecisiete enfermedades tropicales desatendidas incluidas en el listado de la Organización Mundial de la Salud, representa una carga considerable para la salud pública y la economía. De 261 millones de personas que requirieron quimioterapia preventiva para esquistosomiasis en 2013, 92% de ellas vivían en África subsahariana y sólo 12,7% recibieron quimioterapia preventiva. Además, en 2010, la OMS informó que la mortalidad debido a esquistosomiasis podría ser de hasta 280 000 por año tan solo en África.
En mayo de 2012 delegados a la sexagésima quinta Asamblea Mundial de la Salud adoptaron la resolución WHA65.21 que solicitaba la eliminación de la esquistosomiasis y prevé el tratamiento regular de al menos el 75% de los niños en edad escolar en las áreas de riesgo. En la resolución se exhortaba a los estados miembros a intensificar los programas de control de la esquistosomiasis y a iniciar campañas de eliminación dondequiera que fuese posible.
A pesar de ello, en junio de 2015 se indicó que la esquistosomiasis tenía el nivel más bajo de implementación de quimioterapia preventiva en el espectro de las enfermedades tropicales desatendidas. También se la destacó como la enfermedad más carente de progreso. Esto quizás no sea sorprendente dado que era la única enfermedad tropical desatendida (NTD por sus siglas en inglés) con acceso a donaciones de drogas pero sin una coalición de partes interesadas que colaborasen para potenciar el compromiso y la implementación.
En consecuencia, y para asegurar que se cumplan los objetivos de la OMS de 2012 de la hoja de ruta para las NTD y la resolución WHA65.21 de la Asamblea Mundial de la Salud, se ha establecido la Global Esquistosomiasis Alliance (GSA). Diversa y representativa, la GSA busca ser una asociación de países endémicos, instituciones académicas y de investigación, fundaciones y agencias internacionales de desarrollo, organizaciones internacionales, organizaciones no gubernamentales de desarrollo, empresas del sector privado y asociados en actividades de promoción y movilización de recursos. En definitiva, la GSA aboga por una asociación para trabajar para beneficio de los países endémicos al abordar l inequidad en salud y la pobreza rural.

Translated from English version into Spanish by hollidayalicia, through

TRANSLATORS
WITHOUT BORDERS
